# The Prognostic Value of Sarcopenia in Acute Myeloid Leukemia Patients and the Development and Validation of a Novel Nomogram for Predicting Survival

**DOI:** 10.3389/fonc.2022.828939

**Published:** 2022-02-10

**Authors:** Qian Sun, Jialin Cui, Wenjie Liu, Jianyong Li, Ming Hong, Sixuan Qian

**Affiliations:** ^1^Department of Hematology, The First Affiliated Hospital of Nanjing Medical University, Jiangsu Province Hospital, Nanjing, China; ^2^Key Laboratory of Hematology of Nanjing Medical University, Nanjing, China; ^3^Collaborative Innovation Center For Cancer Personalized Medicine, Nanjing Medical University, Nanjing, China; ^4^Department of Rehabilitation Medicine, The First Affiliated Hospital of Nanjing Medical University, Jiangsu Province Hospital, Nanjing, China; ^5^Pukou Chronic Lymphocytic Leukemia (CLL) Center, Pukou Division of Jiangsu Province Hospital, Nanjing, China

**Keywords:** sarcopenia, body composition, acute myeloid leukemia, nomogram, prognosis

## Abstract

**Background:**

Acute myeloid leukemia (AML) occurs frequently in the elderly, of whom the prognosis is dismal. Sarcopenia is a progressive and generalized skeletal muscle disorder associated with an increased possibility of adverse outcomes. This study aims to explore the prognostic value of sarcopenia in AML patients and develop a novel prognostic model.

**Methods:**

A total of 227 AML patients were enrolled. Body composition was assessed by bioelectrical impedance analysis before treatment. Sarcopenia was diagnosed by low muscle quantity. Cox proportional hazard regression model were applied to verify prognostic variables for overall survival (OS) and disease-free survival (DFS). A novel prognostic model of nomogram was developed and validated by ‘R’.

**Results:**

Forty-one (18.1%) patients were defined as sarcopenia. The median age of the sarcopenic group was significantly greater than the non-sarcopenic group (median 70 vs. 64 years, *P* = 0.001). Sarcopenic patients showed significantly less height (*P* = 0.002), weight (*P <*0.001), Body Mass Index (*P <*0.001), Fat Mass (*P* = 0.017), Fat-free Mass (*P <*0.001), Appendicular Skeletal Muscle Mass (*P <*0.001), Skeletal Muscle Index (*P <*0.001), Fat-free Mass Index (*P <*0.001), and hemoglobin level (*P* = 0.025) than the non-sarcopenic ones. Patients in the sarcopenic group also showed a statistically shorter OS and DFS (median OS: 13.7 vs. 55.6 months, *P* = 0.003; median DFS: 12.5 months vs. not reached, *P* = 0.026). ELN high risk [Hazard Ratio (HR): 1.904, 95% Confidence Interval (CI): 1.018–3.562, *P* = 0.044), sarcopenia (HR: 1.887, 95% CI: 1.071–3.324, *P* = 0.028), and reduced-intensity regimens (HR: 3.765, 95% CI: 1.092–12.980, *P* = 0.036) were independent predictors for OS in multivariate analysis. A nomogram for predicting OS was constructed using the above three factors. The *c* index, calibration plots and decision curve analyses (DCA) showed better discrimination, calibration, and net benefits of the nomogram than the ELN model.

**Conclusion:**

Sarcopenia was common and had an inferior prognosis in AML and needs more attention in clinical practice.

## Introduction

Acute myeloid leukemia (AML) is a heterogeneous hematologic malignancy characterized by the clonal expansion of myeloid blasts in the peripheral blood, bone marrow, and/or other tissues. It is the most common form of acute leukemia among adults and accounts for the largest number of annual deaths from leukemias in the world. AML is primarily a disease of older adults, with a median age of 68 years at diagnosis and 59.6% of patients diagnosed at 65 years or older (1). As the population ages, the incidence and mortality seem to be rising. According to the Surveillance, Epidemiology, and End Results (SEER) Cancer Statistics Review, the 5-year overall survival (OS) rate of AML patients over 65 years is only 7.5% (1).

Elderly AML patients are frequently ineligible for or refractory to standard chemotherapy due to advanced age, coexisting conditions, and a high incidence of unfavorable genomic features. Hence, the prognosis of elderly AML patients is dismal. Many reports have focused on the impact of concomitant comorbidities. ﻿The G-8 screening tool was developed and ﻿evaluated based on 364 cancer patients aged >70 years included in a multicenter prospective study, which ﻿suggested good screening classification properties in geriatric oncology. The G-8 consists of seven items from the Mini Nutritional Assessment (MNA) questionnaire and age (2). The Hematopoietic cell transplantation-specific comorbidity index (HCT-CI) was developed with patients who were candidates for allogeneic stem cell transplantation and was then successfully tested in elderly patients with AML (3). Besides, other comprehensive geriatric assessment (CGA) metrics like activities of daily living (ADL), instrumental activities of daily living (IADL), Short Physical Performance Battery (SPPB) have been verified to be prognostic predictors for AML patients (4–6). However, many comorbidities can be well controlled by medications, and above mentioned CGA metrics reflect physical function indirectly.

Recently, sarcopenia has received great attention in the field of clinical nutrition and geriatric medicine. Sarcopenia is a progressive and generalized skeletal muscle disorder (muscle failure) that is associated with an increased possibility of adverse outcomes including falls, fractures, physical disability, and mortality. Low muscle strength, low muscle quantity/quality, and low physical performance are now considered to be important for the diagnosis of sarcopenia, which can be measured by tools directly (7). At present, however, reports about sarcopenia in AML patients are very rare. CGA and nutritional assessment of AML patients are becoming more and more important and worthy of the attention of hematologists. This study aims to explore the incidence, characteristics, and prognostic value of sarcopenia in AML patients and guide the treatment options of elderly patients with AML.

## Materials and Methods

### Patients

A total of 227 AML patients diagnosed from February 2012 to August 2021 in the First Affiliated Hospital of Nanjing Medical University were enrolled in this retrospective study. All patients were diagnosed according to WHO (2016) criteria (8). Exclusion criteria included acute promyelocytic leukemia or another malignancy without remission. Patients must not have previous chemotherapy (except for hydroxyurea) for any myeloid disorder. Patient demographics including age, gender, height, weight, complete blood count, HCT-CI and G-8 score at diagnosis were collected. This study was performed in accordance with the 1964 Declaration of Helsinki and its later amendments and was approved by the Ethics Committee of the First Affiliated Hospital of Nanjing Medical University, Jiangsu Province Hospital. All written informed consents were provided.

### Next-Generation Sequencing

Genomic DNA (gDNA) was extracted from the bone marrow aspirates of each patient using an Autopure extractor (Qiagen, Hilden, Germany). Targeted gene sequencing (TGS) was tested on the Illumina Platforms (Kapa Biosystems, Wilmington, MA, USA) as previously described (9). A total of 42 genes were contained in the TGS panel, including *ASXL1*, *BCOR*, *BCORL1*, *CALR*, *CBL*, *CEBPA*, *CSF3R*, *DNMT3A*, *ETV6*, *EZH2*, *FLT3*, *GATA2*, *IDH1*, *IDH2*, *JAK2*, *KIT*, *KRAS*, *MLL*, *MPL*, *NF1*, *NPM1*, *NRAS*, *PDGFRA*, *PHF6*, *PIGA*, *PPM1D*, *PTPN11*, *RAD21, RUNX1*, *SETBP1*, *SF3B1*, *SH2B3*, *SMC1A*, *SMC3*, *SRSF2*, *STAG2*, *STAT3*, *TET2*, *TP53*, *U2AF1*, *WT1*, and *ZRSR2*.

### Body Composition Analysis

Body composition was assessed with direct segmental multifrequency bioelectrical impedance analysis (DSM-BIA) (BCA-2A, Tongfang Health Technology Co., Ltd, Beijing, PRC) by an experienced nutritionist before treatment. Muscle quantity was documented as Appendicular Skeletal Muscle Mass (ASM), and measurements were normalized to the patient’s height and expressed as Skeletal Muscle Index (SMI; ASM/height^2^). Besides, we collected Body Mass Index (BMI; weight/height^2^), Fat Mass (FM), body fat rate, Fat Mass Index (FMI; FM/height^2^), Fat-free Mass (FFM), and Fat-free Mass Index (FFMI; FFM/height^2^) for further analysis. According to European Working Group on Sarcopenia in Older People 2 (EWGSOP2) cut-off points (7), we here define patients with ASM <20 kg (for men) or <15 kg (for women), and/or SMI <7.0 kg/m^2^ (for men) or <5.5 kg/m^2^ (for women) as sarcopenia.

### Treatment Regimens and Outcome Evaluation

A total of 216 patients received induction therapy, fifty-seven with intensive regimens and 159 with reduced-intensity regimens. Intensive regimens include IA (idarubicin and cytarabine, n = 47), DA (daunorubicin and cytarabine, n = 3), AA (aclarubicin and cytarabine, n = 5), FLAG [fludarabine, high dosage cytarabine and granulocyte colony-stimulating factor (G-CSF), n = 1] and intermediate-dose cytarabine (n = 1). Reduced-intensity regimens consist of DCAG (decitabine, low-dose cytarabine, aclarubicin, and G-CSF, n = 130) (10), DAG (decitabine, low-dose cytarabine, and G-CSF, n = 5), decitabine monotherapy (n = 7), decitabine/azacitidine + venetoclax ± aclarubicin (n = 12), HAG (homoharringtonine, low-dose cytarabine, and G-CSF, n = 3), venetoclax monotherapy (n=1), and hydroxyurea monotherapy (n=1). The choice of intensive or reduced-intensity regimens was dependent on patients’ age and physical performance. Eleven patients received supportive therapy due to frailty or personal preference.

Treatment responses were evaluated according to the National Comprehensive Cancer Network (NCCN) clinical practice guideline of AML (version 3. 2021) (11). Complete remission (CR) is defined as a) bone marrow <5% blasts in an aspirate with spicules and no blasts with Auer rods or persistence of extramedullary disease; b) patients independent of transfusions, with absolute neutrophil count (ANC) >1×10^9^/L (blasts <5%) and platelets (PLT) ≥100×10^9^/L. CR with incomplete hematologic recovery (CRi): meeting all CR criteria and transfusion independence but with persistence of neutropenia (<1×10^9^/L) or thrombocytopenia (<100×10^9^/L). Partial remission (PR) refers to a decrease of at least 50% in the percentage of blasts to 5% to 25% in the bone marrow aspirate and the normalization of blood counts, as noted above. No remission (NR) is regarded as that the blast, peripheral blood counts, and the clinical phenomenon failed to meet the criteria of CR, CRi, or PR. Overall response rate (ORR) includes rates of CR, CRi and PR. Relapse means bone marrow blasts ≥5%; or reappearance of blasts in the blood; or development of extramedullary disease after CR. Overall survival (OS) is measured from the time of diagnosis to death due to any reason or censored at the last follow-up. Disease-free survival (DFS) means the duration from CR until relapse or death or censored at the last follow-up. The last follow-up time was October 1st, 2021.

### Statistics

Continuous variables were described as mean ± standard deviation (SD) (fitting the normal distribution) or median (range) (not complying with the normal distribution), and *t*-test or Mann-Whitney U test was used for comparison between groups. Categorical variables were compared with the Chi-square test, Fisher’s exact test (four grid table), or rank sum test (unidirectional ordered list). The Kaplan-Meier method was used to draw survival curves, and the survival rate comparison between groups was by Log-rank test. Univariable Cox analysis for OS was applied for each parameter separately. Prognostic indicators with a *P*-value of less than 0.1 were included for multivariate Cox model selection. Then, a nomogram to predict the 1-, 2- and 3-year OS rates was developed from the final model to visualize the prognostic value of each risk factor. The predictive accuracy of the nomogram was evaluated by discrimination and calibration. Discrimination was measured *via* the concordance index (*c* index), which quantifies the level of concordance between predicted probabilities and the actual chance of having the event of interest. A *c* index of 0.5 indicates that outcomes are completely random, whereas a *c* index of 1 indicates that the model is a perfect predictor. The calibration curves were used to compare the association between the actual outcomes and the predicted probabilities. Both discrimination and calibration were evaluated using bootstrapping with 1, 000 resamples. The clinical usefulness and benefits of the predictive model were estimated by decision curve analyses (DCA) (12). The nomogram, calibration, and DCA plots were produced using the ‘R’ software [version 4.0.5. R Core Team (2021). R: A language and environment for statistical computing. R Foundation for Statistical Computing, Vienna, Austria. URL https://www.R-project.org/]. All other statistical analyses were performed using SPSS software version 21.0 (IBM Corporation, Armonk, NY, USA), and were plotted by GraphPad Prism 9.0.0 (GraphPad Software, San Diego, CA, USA). All tests were two-sided, and statistical significance was defined as *P*-value <0.05.

## Results

### Patient Characteristics

**Figure 1:**
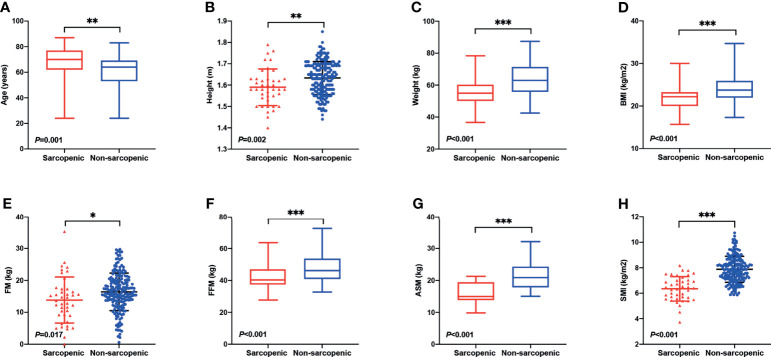
Differences in body composition and demographic indicators between sarcopenic and non-sarcopenic patients. **(A)** The median age of the sarcopenic group was significantly greater than the non-sarcopenic group (median 70 vs. 64 years, *P* = 0.001). Sarcopenic patients showed significantly less **(B)** height (mean 1.59 vs. 1.63 m, *P* = 0.002), **(C)** weight (median 55.0 vs. 63.0 kg, *P <*0.001), **(D)** BMI (median 22.2 vs. 23.8 kg, *P <*0.001), **(E)** FM (mean 13.8 vs. 16.4 kg, *P* = 0.017), **(F)** FFM (median 40.3 vs. 46.2 kg, *P <*0.001), **(G)** ASM (median 15.0 vs. 20.9 kg, *P <*0.001), and **(H)** SMI (mean 6.4 vs. 7.9 kg/m2, *P <*0.001) than the non-sarcopenic ones. BMI, Body Mass Index; FM, Fat Mass; FFM, Fat-free Mass; ASM, Appendicular Skeletal Muscle Mass; SMI, Skeletal Muscle Index. *P < 0.05, **P < 0.01, ***P < 0.001.

Patient characteristics are summarized in **Table 1**. A total of 227 AML patients were enrolled in this study, of whom 109 were males and 118 were females. The median age was 64, with a range from 24 to 87 years. One hundred and fifty-two (67.0%) patients had an HCT-CI score of 0, 51 (22.4%) had an HCT-CI score of 1–2, and 24 (10.6%) had an HCT-CI score of ≥3. Ninety-nine (43.6%) patients were frail and 128 (56.4%) were fit according to ﻿the G-8 screening tool for geriatric oncology (frail: G-8 score ≤14; fit: G-8 score >14). The study cohort consisted of 101 (44.5%) cases of AML with recurrent cytogenetic abnormalities (AML-RCA), 109 (48.0%) cases of AML, not otherwise specified (AML, NOS), 14 (6.2%) cases of AML with myelodysplasia-related changes (AML-MRC), and 3 (1.3%) cases of therapy-related AML (t-AML). Seventy-three (32.1%) patients were classified into low risk due to the 2017 European Leukemia Net (ELN) risk stratification (13), while 71 (31.3%) intermediate risk and 83 (36.6%) high risk. Forty-one (18.1%) patients were defined as sarcopenia according to our criteria, and 186 patients were non-sarcopenic. The percentage of sarcopenia was much higher in patients over 60 years old, which was 21.0% (32/152). The median age of the sarcopenic group was significantly greater than the non-sarcopenic group (median 70 vs. 64 years, *P* = 0.001, **Figure 1A**), suggesting that the muscle quantity of AML patients decreases with the increasing of age. The percentage of frail patients was much higher in sarcopenic patients than in non-sarcopenic ones (80.5% vs. 35.5%, *P <*0.001). Besides, sarcopenic patients showed significantly less height (mean 1.59 vs. 1.63 m, *P* = 0.002), weight (median 55.0 vs. 63.0 kg, *P <*0.001), BMI (median 22.2 vs. 23.8 kg, *P <*0.001), FM (mean 13.8 vs. 16.4 kg, *P* = 0.017), FFM (median 40.3 vs. 46.2 kg, *P <*0.001), ASM (median 15.0 vs. 20.9 kg, *P <*0.001), SMI (mean 6.4 vs. 7.9 kg/m^2^, *P <*0.001), FFMI (median 16.1 vs. 17.5 kg/m^2^, *P <*0.001), and hemoglobin level (mean 74.6 vs. 81.3 g/L, *P* = 0.025) than the non-sarcopenic ones (**Table 1** and **Figures 1B–H**). However, there were no significant differences in gender, body fat rate, FMI, HCT-CI, ELN risk, or common gene mutation rates (*NPM1*, *CEBPA*, *FLT3-ITD*, *FLT3-TKD*, *KIT*, *IDH1*, *IDH2*, *TP53*, *RUNX1*, *ASXL1*, *DNMT3A*, and *GATA2*) between the two groups (**Table 1** and **Supplementary Table 1**).

**Table 1:** Patients’ characteristics.

Characteristics (n = 227)	Total	Sarcopenic (n = 41)	Non-sarcopenic (n = 186)	*P*
**Gender, n (%)**				
male	109 (48.0)	20 (48.8)	89 (47.8)	0.914
female	118 (52.0)	21 (51.2)	97 (52.2)	
**Age (years), median (range)**	64 (24–87)	70 (24–87)	64 (24–83)	**0.001**
**HCT-CI, n (%)**				
0	152 (67.0)	22 (53.7)	130 (69.9)	0.106
1–2	51 (22.4)	14 (34.1)	37 (19.9)	
≥3	24 (10.6)	5 (12.2)	19 (10.2)	
**G-8 frailty screening tool, n (%)**				
fit	128 (56.4)	8 (19.5)	120 (64.5)	**<0.001**
frail	99 (43.6)	33 (80.5)	66 (35.5)	
**Height (m)**	1.63 ± 0.08	1.59 ± 0.09	1.63 ± 0.08	**0.002**
**Weight (kg), median (range)**	60.5 (36.7–87.4)	55.0 (36.7–78.4)	63.0 (42.6–87.4)	**<0.001**
**BMI (kg), median (range)**	23.2 (15.7–34.7)	22.2 (15.7–30.0)	23.8 (17.3–34.7)	**<0.001**
**FM (kg)**	15.9 ± 6.2	13.8 ± 7.2	16.4 ± 5.9	**0.017**
**Body fat rate (%), median (range)**	26.0 (0–48.4)	24.3 (0–48.4)	26.3 (1.3–39.3)	0.445
**FFM (kg), median (range)**	45.4 (27.7–72.6)	40.3 (27.7–63.7)	46.2 (32.7–72.6)	**<0.001**
**ASM (kg), median (range)**	19.8 (9.8–32.2)	15.0 (9.8–21.3)	20.9 (15.0–32.2)	**<0.001**
**SMI (kg/m^2^)**	7.6 ± 1.2	6.4 ± 1.0	7.9 ± 1.0	**<0.001**
**FMI (kg/m^2^)**	6.1 ± 2.5	5.6 ± 3.0	6.2 ± 2.4	0.133
**FFMI (kg/m^2^), median (range)**	17.3 (12.8–23.1)	16.1 (12.8–21.5)	17.5 (13.1–23.1)	**<0.001**
**FM/FFM ratio**	0.36 ± 0.15	0.35 ± 0.20	0.36 ± 0.14	0.807
**WHO classification, n (%)**				
AML-RCA	101 (44.5)	18 (43.9)	83 (44.6)	0.824
AML, NOS	109 (48.0)	21 (51.2)	88 (47.3)	
AML-MRC	14 (6.2)	2 (4.9)	12 (6.5)	
t-AML	3 (1.3)	0 (0)	3 (1.6)	
**^a^** **BM blasts (%), median (range)**	58.0 (6.4–98.8)	57.6 (19.5–92.0)	58.8 (6.4–98.8)	0.182
**WBC (×10^9^/L), median (range)**	6.98 (0.50–260.02)	9.01 (1.33–251.22)	6.53 (0.50–260.02)	0.801
**Hb (g/L)**	80.1 ± 20.4	74.6 ± 15.8	81.3 ± 21.1	**0.025**
**PLT (×10^9^/L), median (range)**	41 (3–1544)	34 (6–645)	44.5 (3–1544)	0.476
**ELN risk, n (%)**				
Low	73 (32.1)	11 (26.8)	62 (33.3)	0.537
Intermediate	71 (31.3)	12 (29.3)	59 (31.7)	
High	83 (36.6)	18 (43.9)	65 (35.0)	
**Therapy, n (%)**				
intensive therapies	57 (25.1)	6 (14.6)	51 (27.4)	0.137
reduced-intensity regimens	159 (70.0)	34 (82.9)	125 (67.2)	
supportive therapy	11 (4.9)	1 (2.5)	10 (5.4)	

a Six patients had a percentage of BM blasts <20%, 5 of them had a percentage of PB blasts ≥20%, and another one had a t(8;21)(q22;q22) and AML1-ETO infusion gene, so all of them could be diagnosed as AML. The complete information of these patients was summarized in **Supplementary Table 2**.

HCT-CI, the Hematopoietic cell transplantation-specific comorbidity index; BMI, Body Mass Index; FM, Fat Mass; FFM, Fat-free Mass; ASM, Appendicular Skeletal Muscle Mass; SMI, Skeletal Muscle Index; FMI, Fat Mass Index; FFMI, Fat-free Mass Index; AML-RCA, AML with recurrent cytogenetic abnormalities; AML, NOS, AML, not otherwise specified; AML-MRC, AML with myelodysplasia-related changes; t-AML, therapy-related AML; BM, bone marrow; WBC, white blood cell; Hb, hemoglobin; PLT, platelet; ELN, European Leukemia Net. Bold values mean the P values were <0.05 and of statistical significance.

### Treatment Responses and Infection Rates

**Figure 2:**
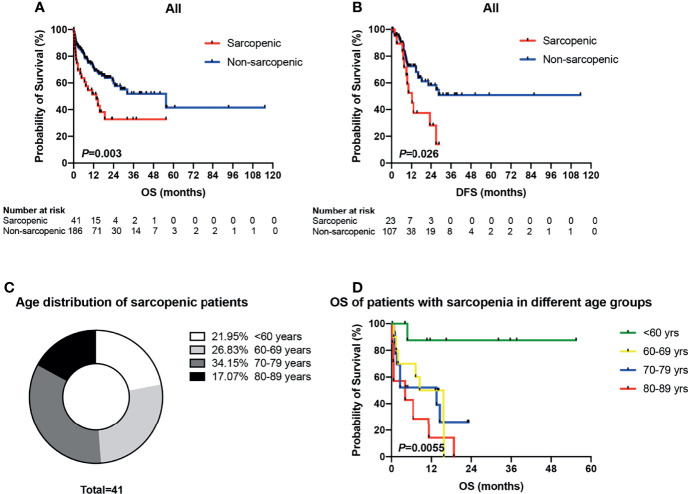
Survival analyses of sarcopenic patients drawn by Kaplan-Meier survival curves. **(A)** Patients in the sarcopenic group showed statistically shorter OS than non-sarcopenic ones (median OS: 13.7 vs. 55.6 months, *P* = 0.003). **(B)** The sarcopenic patients showed significantly shorter DFS than those with non-sarcopenia (median DFS: 12.5 vs. not reached, *P* = 0.026). **(C)** Age distribution of sarcopenic patients. **(D)** Kaplan-Meier plots for overall survival of sarcopenic patients in different age groups (<60 vs. 60–69 vs. 70–79 vs. 80–89 years). OS, overall survival; DFS, disease-free survival.

In the sarcopenic group, 6 (14.6%) patients received intensive therapies, 34 (82.9%) reduced-intensity regimens, and one (2.5%) supportive therapy; and the proportions in the non-sarcopenic group was 27.4% (51/186), 67.2% (125/186) and 5.4% (10/186), respectively. The infection rates of chemotherapy in both groups were summarized in **Table 2**. Sarcopenic patients showed a significantly higher rate of infections with specific sites or reasons than non-sarcopenic ones (65.0% vs. 43.8%, *P* = 0.015), especially a higher rate of lung infection (60.0% vs. 33.0%, *P* = 0.001). While non-sarcopenic patients had a statistically higher rate of febrile neutropenia than sarcopenic ones (44.9% vs. 20.0%, *P* = 0.004). These data suggested that sarcopenic patients were more suitable for reduced-intensity regimens in case of safety. With a median follow-up of 30.7 months, 184 patients were available for response evaluation, of which 33 were in the sarcopenic group and 151 were in the non-sarcopenic group. The CR+CRi rate of non-sarcopenic patients was 59.6%, and the ORR was 72.2%; while the sarcopenic patients gained a CR+CRi rate of 63.7% and an ORR of 75.8%. The CR+CRi, PR, NR rates or ORR suggested no significant differences between the two groups (**Table 3**), while patients in the sarcopenic group showed statistically higher relapse rate (52.2% vs. 26.2%, *P* = 0.014) and shorter OS and DFS than non-sarcopenic ones (median OS: 13.7 vs. 55.6 months, *P* = 0.003, **Figure 2A**; median DFS: 12.5 vs. not reached, *P* = 0.026, **Figure 2B**). The one-year survival rate and DFS rate of sarcopenic patients was 51.3% and 54.0%, of whom the two-year survival rate and DFS rate was only 32.7% and 28.9%.

**Table 2:** Infections rates for patients received chemotherapy.

Adverse events, n(%)	Sarcopenic (n = 40)	Non-sarcopenic (n = 176)	*P*
**Infections with specific sites or reasons**	26 (65.0)	77 (43.8)	**0.015**
sepsis	0 (0)	9 (5.1)	0.215
lung infection	24 (60.0)	58 (33.0)	**0.001**
gum infection	0 (0)	1 (0.6)	1
small intestine infection	1 (2.5)	4 (2.3)	1
anorectal infection	0 (0)	2 (1.1)	1
urinary tract infection	1 (2.5)	1 (0.6)	0.337
soft tissue infection	0 (0)	2 (1.1)	1
**Febrile neutropenia**	8 (20.0)	79 (44.9)	**0.004**

Bold values mean the P values were <0.05 and of statistical significance.

**Table 3:** Treatment responses of patients with sarcopenia and non-sarcopenia.

Outcome (n = 184)	Sarcopenic (n = 33)	Non-sarcopenic (n = 151)	*P*
**CR+CRi, n (%)**	21 (63.7)	90 (59.6)	0.668
**PR, n (%)**	4 (12.1)	19 (12.6)	0.942
**NR, n (%)**	8 (24.2)	42 (27.8)	0.676
**ORR, n (%)**	25 (75.8)	109 (72.2)	0.676
**Relapse, n (%)**	12 (52.2)	28 (26.2)	**0.014**
**Median OS (months)**	13.7	55.6	**0.003**
**1-year survival rate (%)**	51.3	72.1	**0.003**
**2-year survival rate (%)**	32.7	61.8	**0.003**
**Median DFS (months)**	12.5	Not reached	**0.026**
**1-year DFS rate (%)**	54.0	72.5	**0.026**
**2-year DFS rate (%)**	28.9	58.2	**0.026**

CR, complete remission; CRi, CR with incomplete hematologic recovery; PR, partial remission; NR, no remission; ORR, overall response rate; OS, overall survival; DFS, disease-free survival. Bold values mean the P values were <0.05 and of statistical significance.

### Survival Analyses

**Figure 3:**
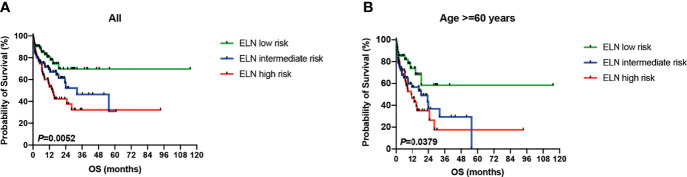
Survival curves of patients stratified by 2017 ELN risk stratification. **(A)** OS curves of all patients were significantly separated according to the ELN risks (*P* = 0.0052). **(B)** OS curves of patients ≥60 years old were significantly separated according to the ELN risks (*P* = 0.0379). ELN, European Leukemia Net.

**Figure 4:**
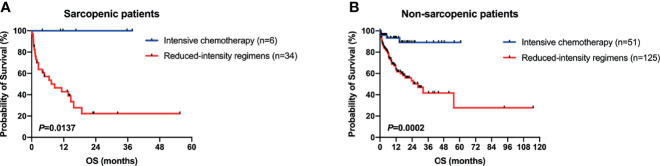
Survival curves of patients stratified by type of chemotherapy. **(A)** In sarcopenic patients, those who received intensive chemotherapy showed a significantly superior OS than reduced-intensity regimens (*P* = 0.0137). **(B)** In non-sarcopenic ones, patients with intensive chemotherapy showed a significantly superior OS than reduced-intensity regimens (*P* = 0.0002).

As mentioned above, there were 41 sarcopenic patients in our study, with 21.95% (9/41) younger than 60 years old, 26.83% (11/41) in their sixties, 34.15% (14/41) in their seventies, and 17.07% (7/41) in their eighties (**Figure 2C**). Survival analysis suggested that sarcopenic patients aged less than 60 years had a significantly superior OS than those ≥60 years (*P* = 0.0055), while patients in the different age groups more than 60 (60–69 vs. 70–79 vs. 80–89) showed no statistical differences in OS (**Figure 2D**). The 2017 ELN risk stratification is also an important prognostic model, OS curves of all patients were significantly separated according to the ELN risks (*P* = 0.0052; **Figure 3A**), so were those of patients ≥60 years old (*P* = 0.0379; **Figure 3B**). Treatment dose intensity was also a significant variable for OS. Those who received intensive chemotherapy showed a significantly superior OS than reduced-intensity regimens in both sarcopenic (*P* = 0.0137, **Figure 4A**) and non-sarcopenic patients (*P* = 0.0002, **Figure 4B**).

Since patients with sarcopenia showed a shorter OS and DFS than non-sarcopenic ones, we next examined the prognostic value of sarcopenia by univariate and multivariate analyses with Cox proportional hazards model. As is shown in **Table 4**, age ≥60 years (*P <*0.001), Hb <100g/L (*P* = 0.077), ELN intermediate risk (*P* = 0.068), ELN high risk (*P* = 0.002), sarcopenia (*P* = 0.004), frailty of G-8 (*P* = 0.001) and reduced-intensity regimens (*P <*0.001) were inferior prognostic factors, and HCT-CI score of 1–2 was a superior prognostic factor for OS in univariate analysis. While ELN high risk [Hazard Ratio (HR): 1.904, 95% Confidence Interval (CI): 1.018–3.562, *P* = 0.044), sarcopenia (HR: 1.887, 95% CI: 1.071–3.324, *P* = 0.028), and reduced-intensity regimens (HR: 3.765, 95% CI: 1.092–12.980, *P* = 0.036) were independent predictors for OS in multivariate analysis. As for DFS, the univariate analysis indicated that age ≥60 years (*P* = 0.078), sarcopenia (*P* = 0.030), frailty of G-8 (*P* = 0.004) and reduced-intensity regimens (*P* = 0.072) were significantly associated with shorter DFS, while only frailty of G-8 (HR: 2.489, 95% CI: 1.332–4.651, *P* = 0.004) was confirmed as the independent predictor in multivariate analysis (**Table 4**).

**Table 4:** Univariate and multivariate analyses for OS and DFS.

Total	OS						DFS					
	Univariate analysis			Multivariate analysis			Univariate analysis			Multivariate analysis		
	*P*	HR	95% CI	*P*	HR	95% CI	*P*	HR	95% CI	*P*	HR	95% CI
**Age (years)**												
<60	Ref.						Ref.					
≥60	**<0.001**	3.974	2.096–7.535				**0.078**	1.782	0.938–3.383			
**Gender**												
Female	Ref.						Ref.					
Male	0.188	1.350	0.864–2.110				0.720	0.889	0.468–1.688			
**Hb (g/L)**												
≥100	Ref.						Ref.					
<100	**0.077**	1.821	0.936–3.544				0.204	1.837	0.719–4.693			
**PLT (×10^9^/L)**												
≥100	Ref.						Ref.					
<100	0.139	1.621	0.854–3.075				0.728	1.148	0.527–2.503			
**ELN risk**												
Low	Ref.			Ref.			Ref.					
Intermediate	**0.068**	1.800	0.957–3.386				0.683	0.850	0.390–1.854			
High	**0.002**	2.622	1.434–4.796	**0.044**	1.904	1.018–3.562	0.171	1.666	0.803–3.459			
**Sarcopenia**												
Non-sarcopenic	Ref.			Ref.			Ref.					
Sarcopenic	**0.004**	2.055	1.254–3.367	**0.028**	1.887	1.071–3.324	**0.030**	2.125	1.075–4.203			
**WBC (×10^9^/L)**												
<100	Ref.						Ref.					
≥100	0.890	1.086	0.340–3.465				0.503	1.637	0.386–6.933			
**HCT-CI**												
0	Ref.						Ref.					
1–2	**0.006**	0.420	0.226–0.783				0.138	1.811	0.826–3.972			
≥3	0.292	0.689	0.345–1.377				0.914	0.936	0.285–3.077			
**G-8 frailty screening tool**												
fit	Ref.						Ref.			Ref.		
frail	**0.001**	2.181	1.390﻿–3.423				**0.004**	2.489	1.332–4.651	**0.004**	2.489	1.332–4.651
**Therapy**												
intensive therapies	Ref.			Ref.			Ref.					
reduced-intensity regimens	**<0.001**	7.536	2.747–20.670	**0.036**	3.765	1.092–12.980	**0.072**	1.893	0.944–3.795			

OS, overall survival; DFS, disease-free survival; Hb, hemoglobin; PLT, platelet; ELN, European Leukemia Net; WBC, white blood cell; HCT-CI, the Hematopoietic cell transplantation-specific comorbidity index; HR, hazard ratio; CI, confidence interval; Ref., reference. The bold values in “Univariate analysis”, mean the P values were <0.1, and the prognostic indicators with a P value of less than 0.1 were included for multivariate Cox model selection. The bold values in “Multivariate analysis”, mean the P values were <0.05 and of statistical significance.

### The Development and Validation of a Novel Nomogram for Predicting Survival

**Figure 5:**
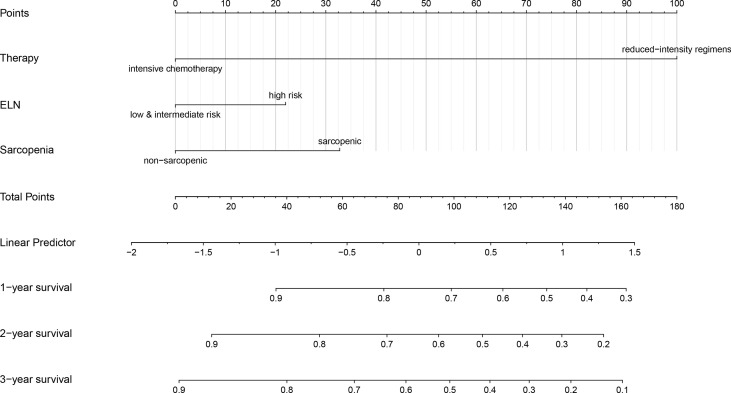
Nomogram for predicting 1-, 2- and 3-year overall survival rates. A new nomogram for predicting OS was constructed by the three significant prognostic factors (therapy, ELN risk, and sarcopenia) determined by the Cox regression analysis. The nomogram was plotted by ‘R, version 4.0.5’. OS, overall survival; ELN, European Leukemia Net.

**Figure 6:**
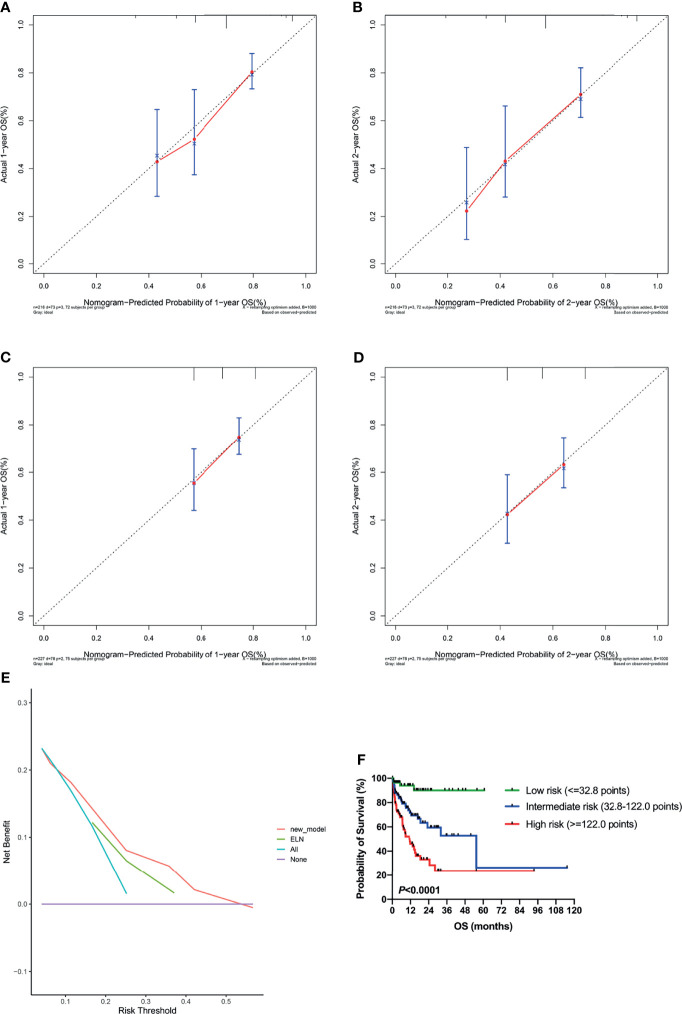
Validation of the nomogram. **(A, B)** Calibration curves showed predicted and actual 1-year and 2-year survival probabilities by the nomogram. The gray diagonal dotted line indicates the perfect correspondence between the observation and prediction. **(C, D)** Calibration curves showed predicted and actual 1-year and 2-year survival probabilities by the 2017 ELN risk stratification (low, intermediate, and high risk). **(E)** Decision curve analysis (DCA) for the nomogram and 2017 ELN risk stratification in prediction of prognosis of AML patients. **(F)** Kaplan-Meier plots for overall survival according to the nomogram score. ELN, European Leukemia Net.

A nomogram for predicting OS was constructed using the three significant prognostic factors (therapy, ELN risk, and sarcopenia) determined by the Cox regression analysis (**Figure 5**). Therapy was the largest contributor in the nomogram, followed by sarcopenia and ELN risk. The calibration plot for the probability of 1- and 2-year OS showed a good linear relationship between prediction by the nomogram and actual observations (**Figures 6A, B**). It also showed a good linear relationship between prediction by the acknowledged 2017 ELN risk stratification (low, intermediate, and high risk) and actual observations (**Figures 6C, D**). The *c* index of the nomogram was 0.69, which is higher than that of the 2017 ELN risk stratification (0.59), indicating that the nomogram was the better fitting model with this cohort.

DCA was performed to compare the clinical usability and benefits of the nomogram with that of the 2017 ELN risk stratification model. Compared to the ELN model, the new nomogram’s DCA curve showed larger net benefits across a range of death risks in our cohort (**Figure 6E**).

Additionally, the cohort was grouped according to the quartile of the total nomogram points of all patients [low risk: the first quartile (1/4) of the whole cohort, nomogram score ≤32.8 points; intermediate risk: nomogram score 32.8–122.0 points; high risk: the last quartile (3/4) of the whole cohort, nomogram score ≥122.0 points], and the OS curves were significantly separated according to the nomogram score (*P <*0.0001; **Figure 6F**), which is more discriminative than the 2017 ELN risk stratification model (**Figure 3A**).

## Discussion

The term sarcopenia was first introduced by Irwin Rosenberg in the 1990s, which was created to describe the loss of muscle mass that accompanies aging (14). The development of sarcopenia has been associated with dysfunction, disability, increased risk of falls and fractures, decreased health-related quality of life, and increased risk of death. The prevalence of sarcopenia in adults with cancer is very high, that is between 11% and 74% in all adults (15), and even higher in the elderly. The accompany with sarcopenia in many cancers is associated with poor prognosis, including head and neck cancer lung cancer, gastric cancer, renal cell carcinoma, diffuse large B-cell lymphoma (16–20), etc. Older adults with cancer face the dual threat of age-related sarcopenia and the pro-inflammatory response of cancer-related cachexia (21). Pathophysiological mechanisms underlying the association between low skeletal muscle mass and poor clinical outcomes in older cancer patients including a) systemic inflammation; b) insulin-dependent glucose handling; c) mitochondrial function; d) protein status and e) pharmacokinetics of anticancer drugs (22).

The incidence of sarcopenia in AML patients and its prognostic value has been seldomly reported. Recently, a study from Gifu University Hospital revealed sarcopenia in 39 (43%) and adipopenia in 35 (39%) in a total of 90 adult AML patients (23). Skeletal muscles and visceral and subcutaneous fat areas were assessed from a single axial slice at the third lumbar (L3) level by computed tomography (CT). Multivariate analysis showed that sarcopenia, together with performance status > 2 and adverse cytogenetic risk were significantly associated with lower OS (23). However, due to the lack of consensus in definitions for sarcopenia and adipopenia by CT, they defined sex specific cutoffs for their population with receiver operator curve (ROC) analysis. Another study from Korea also suggested that sarcopenia and adipopenia assessed by CT scan at the first lumbar vertebra level (L1) can be useful to predict clinical outcomes in patients with AML (24). The current acknowledged 2017 ELN risk stratification by genetics does not contain the physical performance of patients, which is quite important for prognosis. Therefore, this study aims to explore the prognostic value of sarcopenia in Chinese AML patients, and establish a new prognostic model.

In 2010, the European Working Group on Sarcopenia in Older People (EWGSOP) recommended using the presence of both low muscle mass and low muscle function (strength or performance) for the diagnosis of sarcopenia (25). The EWGSOP2 now revised the definition of sarcopenia and considered the role of low muscle strength as a principal determinant (7). Low muscle strength plus low muscle quantity/quality can make the diagnosis of sarcopenia, and patients with low physical performance are considered as severe sarcopenia (7). Muscle quantity can be reported as total body Skeletal Muscle Mass (SMM), ASM, or as muscle cross-sectional area of specific muscle groups or body locations. Magnetic resonance imaging (MRI) and CT are gold standards for the noninvasive assessment of muscle quantity (26). However, these tools are not commonly used in primary care due to the high cost of the equipment, lack of portability, and the need for well-trained personnel (26). Moreover, cut-off points for low muscle mass are not yet well defined for these measurements. Dual-energy X-ray absorptiometry (DXA) is a more widely available instrument to determine muscle quantity non-invasively (27), but different DXA instrument brands do not give consistent results. BIA has been explored for the estimation of SMM or ASM (28). BIA equipment is affordable, widely available, and portable, especially single-frequency instruments. Thus, here we chose DSM-BIA to measure muscle quantity. Besides, physical performance is recommended to be measured by gait speed, SPPB, Timed-up-and-go test (TUG), and 400-meter walk or long-distance corridor walk (400-m walk). The cutoff value of ASM and SMI here was according to the EWGSOP2 criteria (7, 29, 30), which is based on the western population but not Asian cohort, nor cancer patients, therefore, a new criterion of sarcopenia for Asian cancer patients is warranted for further analysis.

This study included both young and old people. Although sarcopenia is common in the elderly, it is an undesirable muscle change throughout a lifetime and may also occur at a young age. This study found that AML patients under 60 years of age can also have sarcopenia (n = 9, accounting for 12.0% of all patients under 60), but in these patients, sarcopenia was not a poor prognostic factor. The stratified analysis suggested that the prognosis of sarcopenia was poor in patients over 60 years of age, which further suggested that the pathophysiological state of young and old AML patients is different.

The prognosis of elderly patients with AML is closely related to physical status and some comorbidities, but among our patients, there was no prognostic effect of HCT-CI on OS or DFS. The G-8 screening tool for frailty showed both statistically prognostic impacts on OS and DFS in univariable Cox analysis, however, it was only a significant predictor for DFS but not OS in multivariate Cox analysis. It may be due to that although some patients had many complications, if properly controlled, they would not affect patients’ chemotherapy tolerance and survival. The muscle quantity/quality is a relatively straightforward way to assess the physical status of patients. Patients with less muscle mass may be more suitable for a weaker regimen; while for some older but not less muscled patients, a more intensive regimen may be more appropriate. The evaluation of sarcopenia before treatment in elderly patients with AML can better help the treatment choosing and prognosis judgment. At present, there are some clinical trials of new drugs for sarcopenia in the world, but whether they can be marketed or improve cancer patients’ performance is not yet known.

The advantage of this study is that it was the first study to report the incidence and characteristics of sarcopenia in Chinese AML patients and to explore the effect of sarcopenia on prognosis. Besides, a new method of the nomogram was used to construct a prognostic model. Furthermore, the *c* index, calibration curve, and DCA curve were used to verify the pros and cons of the new model (compared with the 2017 ELN risk stratification), which is innovative and with important clinical practical value. The shortage of our study is that we did not include patients’ muscle strength or performance because of the retrospective type of this study, and these two variables were not set as principal determinants of sarcopenia at the beginning. We have now been evaluating newly diagnosed AML patients with grip strength for muscle strength, and gait speed and SPPB for physical performance since the year of 2020, however, the total number of patients with complete records was relatively small, so we did not contain muscle strength and performance in this study. Besides, the muscle mass was measured by the BIA method, which is not the gold standard for the noninvasive assessment of muscle quantity. In addition, the patient cohort was relatively small, and the new prognosis model was not verified internally and externally. Given the above issues, further studies are needed to examine the association between sarcopenia and AML.

## Conclusions

In summary, our study firstly reported the incidence and characteristics of sarcopenia in a retrospective cohort of 227 Chinese patients with AML, and sarcopenia was shown to have an inferior prognostic impact on these patients. A new nomogram model including therapy, ELN risk, and sarcopenia was established and validated to evaluate the prognosis of AML patients. Studies with a larger sample size are needed to validate this model.

## Data Availability Statement

According to national legislation/guidelines, specifically the Administrative Regulations of the People’s Republic of China on Human Genetic Resources (http://www.gov.cn/zhengce/content/2019-06/10/content_5398829.htm, http://english.www.gov.cn/policies/latest_releases/2019/06/10/content_281476708945462.htm), no additional raw data is available at this time. Data of this project can be accessed after an approval application to the China National Genebank (CNGB, https://db.cngb.org/cnsa/). Please refer to https://db.cngb.org/, or email: CNGBdb@cngb.org for detailed application guidance. The accession code CNP0002608 should be included in the application.

## Ethics Statement

The studies involving human participants were reviewed and approved by the Ethics Committee of the First Affiliated Hospital of Nanjing Medical University, Jiangsu Province Hospital. The patients/participants provided their written informed consent to participate in this study.

## Author Contributions

Conceptualization, MH and SQ. Methodology, QS and JC. Validation, WL and MH. Formal analysis, QS and JC. Investigation, QS and JC. Data curation, WL. Writing—original draft preparation, QS and JC. Writing—review and editing, WL, MH, and JL. Visualization, QS. Supervision, JL and SQ. Project administration, SQ. Funding acquisition, SQ. All authors have read and agreed to the published version of the manuscript.

## Funding

This research was funded by National Natural Science Foundation of China, grant number 81870119 and 82170153.

## Conflict of Interest

The authors declare that the research was conducted in the absence of any commercial or financial relationships that could be construed as a potential conflict of interest.

## Publisher’s Note

All claims expressed in this article are solely those of the authors and do not necessarily represent those of their affiliated organizations, or those of the publisher, the editors and the reviewers. Any product that may be evaluated in this article, or claim that may be made by its manufacturer, is not guaranteed or endorsed by the publisher.
