# Supplementary material for: The Prognostic Value of Sarcopenia in Acute Myeloid Leukemia Patients and the Development and Validation of a Novel Nomogram for Predicting Survival
Source: Front Oncol. 2022 Feb 10;12:828939. doi: 10.3389/fonc.2022.828939 (PMC8866858; doi:10.3389/fonc.2022.828939)
Supplement: Supplementary file 1 [file Table_1.docx]

Supplementary Material

# Supplementary Table 1. The characteristics of different gene mutations in sarcopenic and non-sarcopenic AML patients

| **Characteristics (n=227)** | **Total** | **Sarcopenic (n=41)** | **Non-sarcopenic (n=186)** | ***P*** |
| --- | --- | --- | --- | --- |
| ***NPM1* (n=217), n (%)** |  |  |  |  |
| mutated | 40 (18.4) | 6 (15.8) | 34 (19.0) | 0.644 |
| wild type | 177 (81.6) | 32 (84.2) | 145 (81.0) |  |
| ***CEBPA* (n=217), n (%)** |  |  |  |  |
| biallelic mutated | 25 (11.5) | 3 (7.9) | 22 (12.3) | 0.441 |
| non-biallelic mutated | 192 (88.5) | 35 (92.1) | 157 (87.7) |  |
| ***FLT3-ITD* (n=217), n (%)** |  |  |  |  |
| mutated | 33 (15.2) | 3 (7.9) | 30 (16.8) | 0.167 |
| wild type | 184 (84.8) | 35 (92.1) | 149 (83.2) |  |
| ***FLT3-TKD* (n=217), n (%)** |  |  |  |  |
| mutated | 10 (4.6) | 0 (0) | 10 (5.6) | 0.216 |
| wild type | 207 (95.4) | 38 (100) | 169 (94.4) |  |
| ***KIT* (n=217), n (%)** |  |  |  |  |
| mutated | 9 (4.1) | 0 (0) | 9 (5.0) | 0.365 |
| wild type | 208 (95.9) | 38 (100) | 170 (95.0) |  |
| ***IDH1* (n=208), n (%)** |  |  |  |  |
| mutated | 25 (12.0) | 2 (5.7) | 23 (13.3) | 0.208 |
| wild type | 183 (88.0) | 33 (94.3) | 150 (86.7) |  |
| ***IDH2* (n=208), n (%)** |  |  |  |  |
| mutated | 27 (13.0) | 7 (20.0) | 20 (11.6) | 0.175 |
| wild type | 181 (87.0) | 28 (80.0) | 153 (88.4) |  |
| ***TP53* (n=208), n (%)** |  |  |  |  |
| mutated | 26 (12.5) | 4 (11.4) | 22 (12.7) | 0.834 |
| wild type | 182 (87.5) | 31 (88.6) | 151 (87.3) |  |
| ***RUNX1* (n=209), n (%)** |  |  |  |  |
| mutated | 25 (12.0) | 7 (20.0) | 18 (10.3) | 0.108 |
| wild type | 184 (88.0) | 28 (80.0) | 156 (89.7) |  |
| ***ASXL1* (n=209), n (%)** |  |  |  |  |
| mutated | 37 (17.7) | 10 (27.8) | 27 (15.6) | 0.082 |
| wild type | 172 (82.3) | 26 (72.2) | 146 (84.4) |  |
| ***DNMT3A* (n=208), n (%)** |  |  |  |  |
| mutated | 38 (18.3) | 3 (8.6) | 35 (20.2) | 0.104 |
| wild type | 170 (81.7) | 32 (91.4) | 138 (79.8) |  |
| ***GATA2* (n=208), n (%)** |  |  |  |  |
| mutated | 7 (3.4) | 0 (0) | 7 (4.0) | 0.604 |
| wild type | 201 (96.6) | 35 (100) | 166 (96.0) |  |

**Supplementary Table 2.**  The complete information of six patients with a percentage of BM blasts <20%

| **Patient ID** | **Gender** | **Age** | **WHO classification** | **BM blasts (%)** | **PB blasts (%)** | **Cytogenetics** | **Gene mutations** | **Sarcopenia** |
| --- | --- | --- | --- | --- | --- | --- | --- | --- |
| 127 | F | 77 | AML-RCA | 17.6 | 27 | 46, XX, del(9)(q21) [10] | *RUNX1* | Non-sarcopenic |
| 156 | M | 51 | AML-MRC | 14.4 | 27 | 47, XY, +8 [6]/46, XY [4] | *GATA2*, *JAK2*, *MPL*, *U2AF1* | Non-sarcopenic |
| 168 | M | 70 | AML-RCA | 6.4 | 38 | 46, XY [20] | *CEBPA* biallelic mutation, *TET2* | Non-sarcopenic |
| 185 | F | 72 | AML, NOS | 18 | 20 | 46, XX [20] | *TET2* | Non-sarcopenic |
| 193 | M | 74 | AML-RCA | 19.5 | 23 | 46, XY [7] | *DDX41*, *DNMT3A*, *PHF6*, *RUNX1* | Sarcopenic |
| 212 | F | 47 | AML-RCA | 10.4 | 18 | 46, XX, t(8;21)(q22;q22) [10] | *CBL*, *NRAS* | Non-sarcopenic |

Abbreviations: F: Female; M: Male; AML-RCA: AML with recurrent cytogenetic abnormalities; AML-MRC: AML with myelodysplasia-related changes; AML, NOS: AML, not otherwise specified; BM: bone marrow; PB: peripheral blood.
